# Effects of the combined extracts of *Herba Epimedii* and *Fructus Ligustrilucidi* on airway remodeling in the asthmatic rats with the treatment of budesonide

**DOI:** 10.1186/s12906-017-1891-0

**Published:** 2017-08-01

**Authors:** Xiufeng Tang, Honglei Nian, Xiaoxi Li, Yan Yang, Xiujuan Wang, Liping Xu, Haotian Shi, Xinwei Yang, Renhui Liu

**Affiliations:** Beijing Key Lab of TCM Collateral Disease Theory Research, School of Traditional Chinese Medicine, Capital Medical University, No.10 Xitoutiao, Youanmenwai, Fengtai District, Beijing, 100069 China

**Keywords:** Herba Epimedii, Fructus Ligustri Lucidi, Airway remodeling, Asthma model

## Abstract

**Background:**

Asthma is characterized by chronic airway inflammation, leading to structura1 changes in the airway, collectively termed airway remodeling. Airway remodeling is thought to contribute to airway hyper responsiveness and irreversible airflow limitation. The combination of *Herba Epimedii* (HE) and *Fructus Ligustri Lucidi* (FLL) decoction and the systemic administration of glucocorticoids (GC) had a synergistic inhibitory action on airway inflammation in the asthmatic model rats. However, the effects of the combination on airway remodeling have not been studied and compared. In the present study, we investigated the effects of the co-administration of combined extracts of HE and FLL with inhaled GC (budesonide) on airway remodeling in the rat asthmatic model induced by ovalbumin (OVA).

**Methods:**

Male Sprague-Dawley rats were sensitized to intraperitoneal OVA followed by repetitive OVA challenge for 7 weeks. Treatments included extracts of HE and FLL (Extracts for short, 100 mg/kg by gastric perfusion), budesonide (1 mg budesonide suspension in 50 ml sterile physiological saline, 3 rats in an ultrasonic nebulizer by nebulized inhabation with a flow of 1.6 ml/min for 30 min), and co-administration of extracts of HE and FLL with budesonide (Co-administration for short) for 4 weeks. Lung histomorphometry and bronchoalveolar lavage fluid (BALF) cell count were assessed 24 h after the final OVA challenge. Levels of interleukin (IL)-4, IL-5 and IgE were measured by ELISA. Expressions of Collagen I and Collagen III were tested by immunohistology. Expressions of transforming growth factor (TGF) -β1, TGF-β2 and Smads mRNA were measured by quantitative real-time PCR.

**Results:**

Extracts, budesonide and Co-administration significantly reduced allergen-induced increases in the serum levels of IL-4, IL-5 and IgE, the number of eosinophils in BALF, goblet cell hyperplasia, Collagen III integral optical density (IOD) and the mRNA expression of TGF-β2 and Smad2. Extracts and Co-administration could depress the IOD level of Collagen I and the positive area of Collagen I and Collagen III. Budesonide and Co-administration significantly alleviated the thickening of airway wall. Only Co-administration significantly decreased collagen deposition according to the morphometry of Masson’s-stained lung sections, the thickening of airway smooth muscle layer, the number of lymphocytes in BALF and the mRNA expression of TGF-β1 and Smad3, and this was associated with a significant increase in levels of Smad7 mRNA.

**Conclusions:**

The findings suggested that the combination of budesonide and the herbal extracts had a better synergistic effect on airway remodeling in OVA-reduced asthma rats than the single use of budesonide.

## Background

Bronchial asthma has become one of the most common health problems in the world, especially within industrialized societies [1, 2]. Increasing evidences suggest that asthma is a chronic inflammatory disorder of the airways in which many cells and cellular elements play important roles and is characterized by the infiltration of eosinophils and dominant in helper T cells (Th) 2 cytokines [3–5]. The frequent occurrence of injury and repair initiated by chronic inflammation could lead to structura1changes in the airway, collectively termed airway remodeling. Airway remodeling is characterized by airway wall thickening, subepithelial fibrosis, increased smooth muscle mass, angiogenesis and increased mucous glands [6, 7]. Generally, airway remodeling is thought to contribute to airway hyper-responsiveness and irreversible airflow limitation.

All recent consensus statements on asthma advocate aggressive treatment of airway inflammation [8, 9]. Glucocorticoids (GCs), especially inhaled GCs, remain the cornerstone of asthma management because of the most effective anti-inflammatory effect, tolerability and rapid onset of action [10]. Prolonged low dose administration of inhaled GC is generally considered safe, although there are considerable concerns about the long-term effects of GC among some consumers, particularly children and the GCs-resistant asthmatic patients [11]. Severe asthma has a distinct pathophysiology including airway remodeling that contributes to the decreased effectiveness of standard therapy. However, when moderate or high doses are required to control symptoms, adverse effects such as growth stunting in children [12], suppression of the hypothalamic-pituitary-adrenal (HPA) axis [13, 14], and osteopenia [15] may be observed. For these reasons, effective therapies that are targeted at severe asthma and that can inhibit asthma airway remodeling are needed.

Traditional Chinese medicine (TCM) has a unique advantage in relieving and curing asthma [16, 17]. There are a number of anti-asthma herbal formulas recorded in TCM literatures and used in practice, but evidence-based researches into their efficacy and mechanisms of efficacy are still in their infancy. According to the theory of TCM, kidney-*yang* deficiency syndrome is one of the most common syndromes seen in asthmatics and may run through the entire process of asthma [18–20]. TCM doctors often follow the principle of strengthening kidney-*yang* to treat asthma. Because the syndrome of the asthmatic patient with long-term or high-dose administration of GC is similar to the clinical signs of the kidney-*yang* deficiency syndrome [21, 22], the herbal medicines of replenishing kidney were often use to prevent the adverse effects of GC when GC was prescribed to treat asthma for a long term or at a high dose by TCM doctor.

Professor *Huiguang Xu,* a famous doctor of TCM, has used the decoction of *Herba Epimedii* (HE) and *Fructus Ligustri Lucidi* (FLL) to treat kidney-*yang* deficiency syndrome of asthma, or to combine with GC for the treatment of asthma for almost 50 years [23, 24]. HE and FLL, documented in Chinese ancient medicinal literatures, have strong actions of replenishing kidney-*yang* and kidney-*yin*, respectively [25, 26]. In our previous researches, we established an asthma model in rats by repetitive ovalbumin (OVA)-challenge which replicated many of the features of the human disease asthma with a high degree of fidelity, and demonstrated that the decoction of HE and FLL in combination with administration of systemic GC (dexamethasone) had a better anti-inflammatory effect on the asthmatic model rats, and could prevent the inhibition of HPA axis and loss of bone mass, compared with the administration of dexamethasone [27–29]. However, we found that the effect of dexamethasone on the mRNA expression of glucocorticoids receptor (GR) isoforms in lung tissue was not consistent with the protein content of GR isoforms, suggesting that inhaled GC was less likely to produce GC resistance than systemic administration of GC. So we choose budesonide as a representative drug for inhaled GC to research the effects of EL on the asthmatic rats with budesonide intervention. This study aimed to compare the effects of the extracts of HE and FLL, inhaled GC, and the combination o f the herbal extracts with inhaled GC on airway inflammation and remodeling in asthmatic rat, and provide basis for the combination of Chinese and Western medicine in the treatment of asthma.

## Methods

### Preparation of herbal extracts

HE, the dried leaf of *Epimediium brevicornu Maxin.* and FLL, the dried mature seed of *Ligustrum lucidum Ait.*, were purchased from Beijing *Tongrentang* pharmaceutical Co. Ltd., China, and authenticated by an expert herbalist at Capital Medical University. They were stored in a dry and sealed container at 4 °C. Preparation of HE and FLL extracts were performed according to the methods described before [30].

### Animals

Fifty male Sprague-Dawley rats, weighing 120 to130 g with the average age of four or five weeks, were purchased from Vital River Laboratory Animal Technology Co. Ltd. (Beijing, China). The experiment complied with the Animal Management Rule of the Ministry of Public Health, China, and the experimental protocol was approved by the Animal Care Committee of Capital Medical University, Beijing, China. All the animals were cared for in the Experimental Animal Center of Capital Medical University. During the whole experiment, the animals were housed in stainless cages (three rats per cage) at conventional controlled conditions (temperature of 23 ± 2 °C, relative humidity of 50 ± 10%, 12-h light-dark cycle). They were allowed for free access to the standard laboratory food and tap water.

### Experimental protocol

After acclimatization for 7 days, the rats were randomly assigned into the following five groups (*n* = 10 *per* group): normal control group (Control), asthma model group (Asthma), budesonide group (Budesonide), group of herbal extracts (Extracts), and group of co-administration of budesonide with herbal extracts (Co-administration).

OVA sensitization and challenge protocols were performed according to the methods of Yang et al. [31] with certain modifications as described below. All the rats with the exception of those in the control group were actively sensitized with an intraperitoneal (*ip*) injection and subcutaneous injection of 1 mg OVA (Grade II; Sigma, USA) and 100 μg aluminum hydroxide (Sigma) in 1 ml sterile physiological saline. After seven days, the same procedure was repeated. After another seven days, the OVA-sensitized rats were exposed to 1% aerosolized OVA (1 g OVA in 100 ml sterile physiological saline in an ultrasonic nebulizer) for 30 min once a day. Three weeks later, the OVA-sensitized rats were challenged twice a week and treated every day. Rats in the budesonide and co-administration groups were exposed to budesonide suspension for inhalation (AstraZeneca Pty Ltd., AUS; 1 mg budesonide suspension in 50 ml sterile physiological saline, three rats in an ultrasonic nebulizer with a flow of 1.6 ml/min) for 30 min. Rats in the Extracts and Co-administration groups were administered with the combined extracts of HE and FILL via oral gavage at the dose of 100 mg/kg body weight. The rats in the normal control group and asthma model group were given with the same volume of distilled water. On the 64th day, all the rats were anesthetized with 25% ethyl carbamate (4 mL/kg, *ip*).

### Cell count of BALF cytospin

The right lungs were lavaged 3 times with 3 mL, 3 mL and 4 mL ice-cold saline using a tracheal cannula and a 5 mL polyethylene syringe. The cell-debris pellets of bronchoalveolar lavage fluid (BALF) samples were collected after centrifugation (500 rpm, 5 min, and 4 °C). A differential cell count was performed on cytospin by Wright-Giemsa staining with the Nikon ECLIPSE 80i biomicroscope and NIS-Elements BR 3.2 image analysis system (Nikon, Japanese). The number of lymphocytes and eosinophils in 200 cells was counted.

### Lung histopathology and morphometry

The middle lobe of the left lungs was removed and fixed in 4% neutral-buffered paraformaldehyde solution in PBS (pH 7.4) for 24 h, embedded in paraffin and then routinely processed. Lung tissue sections (4 μm) were stained with hematoxylin and eosin (H&E), Masson’s trichrome and periodic-acid schiff (PAS) stain. A minimum of 3 bronchi (luminal diameter, 150–500 μm) were analyzed *per* rat for various parameters using an image-analyzing computer system (NIS-Elements BR 3.2; Nikon, Japan).

H&E-stained lung sections were used mainly for the assessment of peribronchial inflammation and airway remodeling. According to the method of Ming Chen et al. [32], 3 bronchioles with 150–200 μm inner diameter were selected and counted in each slide. The perimeter of basement membrane (Pbm), total area of airway wall (Wat) and area of smooth muscle (Wam) were measured and the average was calculated. Pbm was used for normalization of Wat and Wam. Then we used the ratios of Wat to Pbm (Wat/Pbm) and Wam to Pbm (Wam/Pbm) to evaluate airway remodeling.

Masson’s trichrome-stained sections were used for identification of subepithelial collagen [33, 34]. Briefly, the epithelial basement membranes (diameter ≥ 250 μm) were selected, and the basement membrane perimeter and collagen fiber area (stained in blue) beneath the basement membrane in 20 μm depth were measured. The mean score of the fibrotic area divided by the basement membrane perimeter in every rat was calculated.

PAS-stained sections were used for observing the goblet cells [35, 36]. The length of the epithelial basement membrane of the bronchus of ≥500 μm was selected, and the number of PAS-positive goblet cells was counted standardized to Pbm to account for variations in bronchiole diameters. The area of goblet cells within the epithelial lining area of 100 μm^2^ was analyzed for each airway.

### Enzyme linked immunosorbent assy (ELISA)

Blood samples were collected and separated simultaneously using a centrifuge (Biofuge 15R, Heraeus Sepatech, USA), and serum was collected finally and stored at −80 °C prior to assay. The serum levels of IL-4, IL-5 and IgE were measured using rat ELISA kits (Cusabio Biotech CO., Ltd., Wuhan, China) by enzyme-linked immunosorben assay (Immunodiagonstic System Ltd., Boldon, UK), and absorbance was read using ELISA reader (Thermo, USA) at 450 nm.

### Immunohistochemical analysis

Collagen I and Collagen III was identified in paraffin-embedded sections of the lung tissue by immunohistochemical staining with anti-Collagen I antibody or anti-Collagen III antibody (Abcam, Cambridge, UK) overnight at 4 °C at a concentration of 1:100 following by standard biotin-streptavidin-peroxidase immunostaining using a streptavidin-peroxidase kit (Beijing Zhongshan Goldenbridge Biotechnology, Beijing, China) following the instructions provided by the manufacturer. Staining was completed by incubation with diaminobenzidine chromogen solution at room temperature. All measurements were performed with the Nikon ECLIPSE 80i biomicroscope and NIS-Elements BR 3.2 image analysis system (Nikon, Japanese). Three random images within a lung sample from 3 transverse sections were taken, and further analyzed by using zoomed-in field at 400 × magnification. We measured the integral optical density (IOD) and the area of Collagen I or Collagen III-positive cells under each examined field, and calculated the average number as the final result of this sample.

### Quantitative real-time PCR (qPCR)

Total RNA was isolated from the lung tissue using TRIzol reagent (Invitrogen, CA, USA) according to the manufacturer’s recommendations. Total RNA (2 μg) was reverse-transcribed using the Superscript First Strand synthesis system (Invitrogen, CA, USA) to generate complementary DNA (cDNA). The qPCR amplification was performed using the SYBR-green detection of PCR products in real time with an ABI-7500 Sequence Detection System (Applied Biosystems, Foster City, CA, USA). The primers used in the qPCR analysis are presented in Table 1. The PCR program was performed for 40 cycles with each cycle consisting of 30 s of denaturation at 95 °C, 3 s of annealing at 95 °C, and 30 s of extension at 60 °C. Gene expression was quantified by means of the comparative Ct method (^ΔΔ^Ct) and the relative quantification (RQ) was calculated as 2^−ΔΔCt^. Relative mRNA levels of TGF-β1, TGF-β2, Smad2, Smad3 and Smad7 were examined and normalized to β-actin mRNA expression in each sample. The melting curves for each PCR reaction were generated to ensure the purity of the amplification product. A no-template negative control was included in each experiment.

**Table 1:** Primers used for qPCR analysis

Primer	Forward primer	Reverse primer
TGF-β1	TGAGTGGCTGTCTTTTGACG	TGGGACTGATCCCATTGATT
TGF-β2	GCGAGCGAAGCGACGAGGAG	TGGGCGGGATGGCATCAAGGTA
Smad2	CGATGCTCAAGCATGTCCTA	CGCTCTGGGTTTTGACTAGC
Smad3	TTTAGCATTCTGCCGCTTTT	TGCCCCAGTTTTACCAAGTC
Smad7	CCAACTGCAGACTGTCCAGA	CAGGCTCCAGAAGAAGTTGG
β-actin	AGCCATGTACGTAGCCATCC	ACCCTCATAGATGGGCACAG

### Statistical analysis

Results of all measurements were presented as means ± standard deviation (SD). The data analysis was performed using the SPSS 13.0 (SPSS Inc., Chicago, USA). All of the data were tested for normality using the Kolmogorov-Smirnov test, and passed. A one-way analysis of variance (ANOVA) was performed to determine whether there were statistically significant differences (*P* < 0.05) among the experimental groups. The Duncan’s multiple range post-hoc test was used for comparisons between individual groups and to determine which means differed statistically significantly (*P* < 0.05).

## Results

### Effects of budesonide and the herbal extracts on airway remodeling in H&E sections

**Fig. 1:**
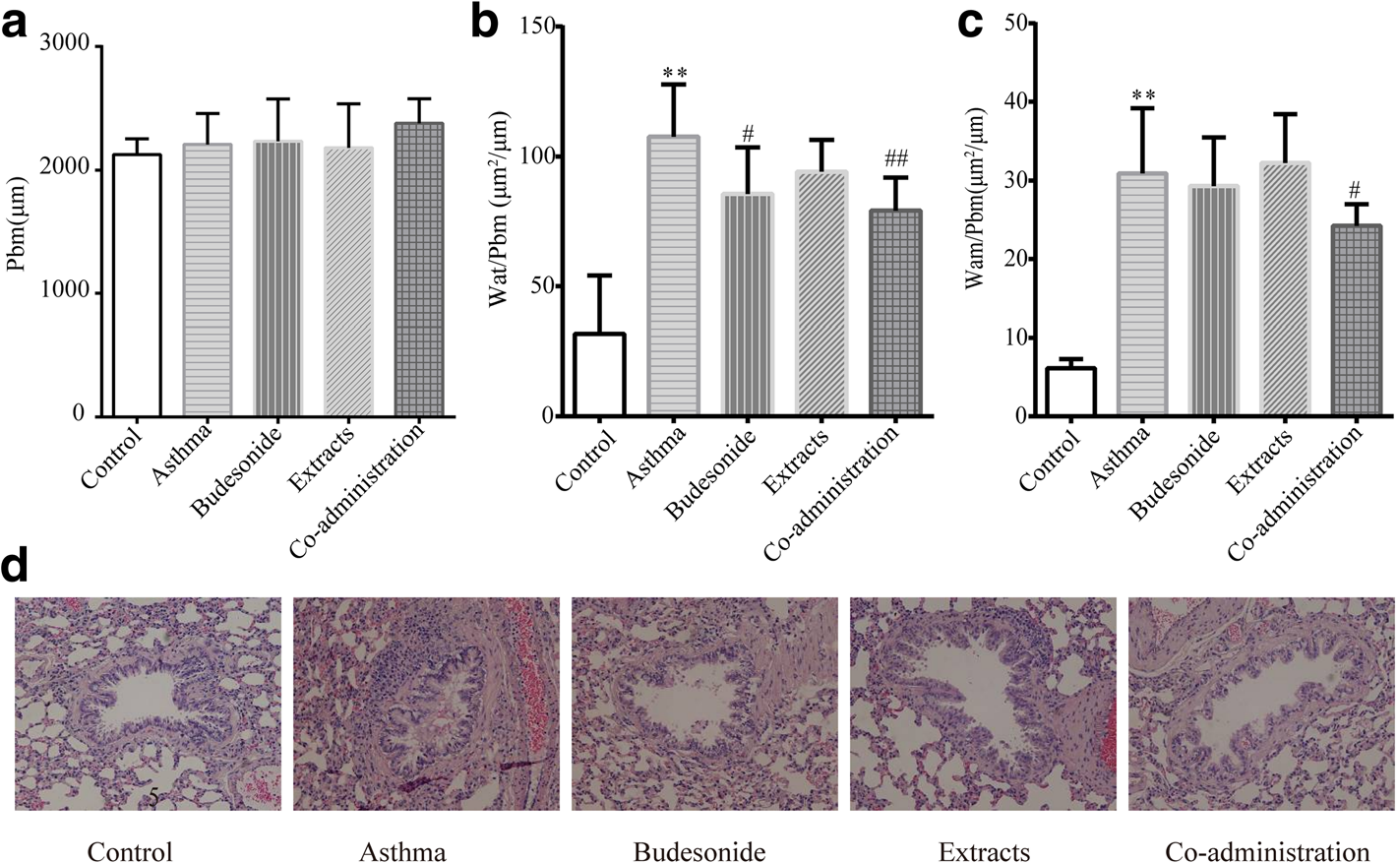
Effects of budesonide and the herbal extracts on airway remodeling. Pbm (**a**), Wat/Pbm (**b**), and Wam/Pbm (**c**) in H&E-stained lung sections. (**d**) Representative photomicrographs of H&E-stained lung sections from each group (×400); Mean ± SD, *n* = 10. ^**^ *P* < 0.01compared to normal control group; ^#^ *P* < 0.05 and ^##^ *P* < 0.01compared to asthma model group

To determine whether budesonide and the herbal extracts were involved in the development of airway remodeling, we evaluated the peribronchial cellular infiltration and airway smooth muscle thickness in all experimental rats. Representative sections of each group were stained with H&E (Fig. 1d). As shown in Fig. 1d, no inflammation, mucosal edema and epithelial lesions were observed in the control group, whereas OVA-induced asthma rats developed severe inflammation, mucosal edema and epithelial lesions, which included interstitial infiltrates and a large number of lymphocyte and eosinophil infiltration. After treating with budesonide, the herbal extracts or the Co-administration, mild to moderate inflammation, mucosal edema and epithelial lesions were observed. According to results of the morphometry of H&E sections, we calculated the ratios of Wat/Pbm and Wam/Pbm to evaluate airway remodeling. Figure 1b showed that the OVA-induced asthma rats presented thicker airway walls than the normal control group (*P* < 0.01) after correction for airway basement perimeter (Wat/Pbm). Budesonide and Co-administration groups could effectively reduce airway wall thickening (Wat/Pbm; *P* < 0.05 and *P* < 0.01, respectively). For Wam/Pbm (Fig. 1c), the asthma model group had an increased smooth muscle layer compared with the normal control group (*P* < 0.01), and only the Co-administration group could reduce myocyte hyperplasia (*P* < 0.05). There were no significant differences in Wat/Pbm and Wam/Pbm between the groups of Budesonide and Extracts.

### Effects of budesonide and the herbal extracts on collagen deposition and goblet cell hyperplasia

**Fig. 2:**
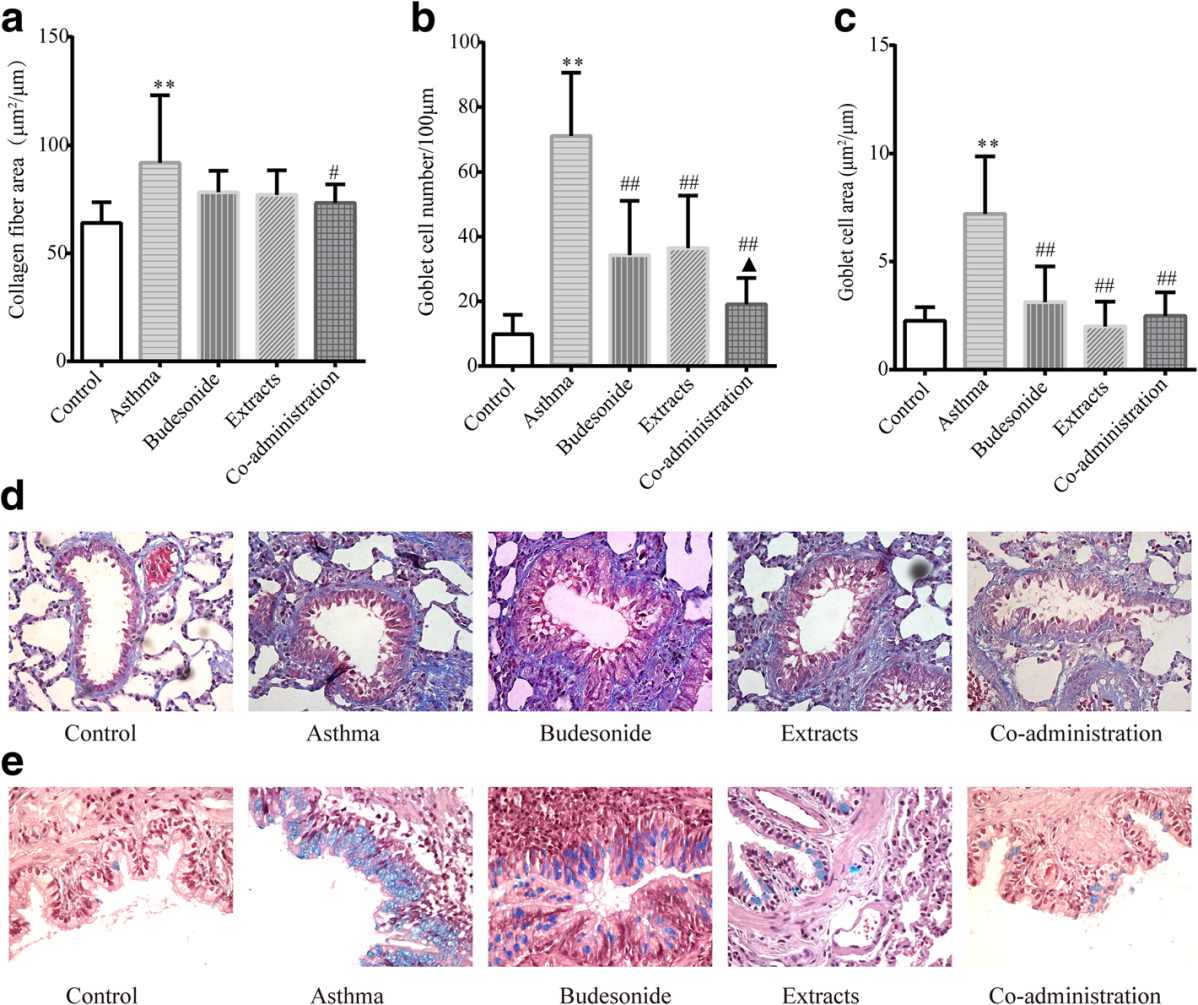
Effects of budesonide and the herbal extracts on collagen deposition and goblet cell hyperplasia. (**a**) Collagen fiber area in Masson’s trichrome-stained sections. (**b**) The number of the goblet cells in PAS-stained lung sections. (**c**) The area of the goblet cells in PAS-stained lung sections. (**d**) Representative photomicrographs of Masson’s trichrome-stained sections from each group (×400). (**e**) Representative photomicrographs of PAS-stained lung sections from each group (×400). Mean ± SD, *n* = 10. ^**^ *P* < 0.01 compared to normal control group; ^#^ *P* < 0.05 and ^##^ *P* < 0.01 compared to asthma model group; ^▲^ *P* < 0.05 compared to budesonide group

Mucus hypersecretion and collagen hyperplasia, which are the main pathological features in asthma and contributes significantly to airflow limitation, are accompanied by Collagen deposition and goblet cell hyperplasia. Collagen deposition within the rat airway wall was performed by the Masson’s trichrome stainning. Figure 2a showed that the area of peribronchial collagen fiber was significantly greater in the asthma model group than that in the normal control group (*P* < 0.01). Only the Co-administration group could significantly decrease the area of collagen deposition, compared with the asthma group (*P* < 0.05). PAS-stained lung sections were performed to evaluate goblet cell hyperplasia in the airway epithelium, which is a cardinal feature of bronchial asthma. As shown in Fig. 2e, goblet cell hyperplasia was observed in the asthma model group, but not in the control group. The number and area of goblet cells in the epithelium greatly increased following repeated OVA-challenge (*P* < 0.01), and showed the hypertrophic features. Compared with the asthma model group, a significant decrease was noticed in airway secretion in all three treatment groups (all *P* < 0.01), which indicated that budesonide and herbal extracts markedly reduced goblet cell hyperplasia in airways. There was a significant difference in goblet cell number between the budesonide group and the co-administration group (*P* < 0.05). These data indicated that the combination of budesonide and herbal extracts had a better effect in reducing goblet cell hyperplasia.

### Effects of budesonide and the herbal extracts on airway inflammation

**Fig. 3:**
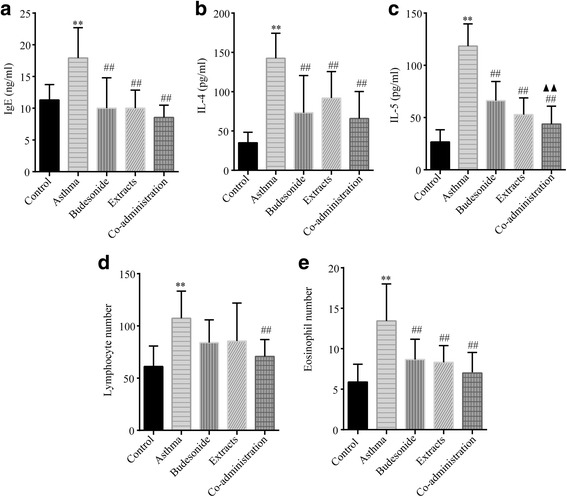
Effects of budesonide and the herbal extracts on airway inflammation. The serum levels of IL-4 (**a**), IL-5 (**b**) and IgE (**c**) were assayed by ELISA. Lymphocytes (**d**) and eosinophils (**e**) were count on BLAF cytospin by Wright-Giemsa staining. The serum levels of IL-4, IL-5 and IgE and the number of lymphocytes and eosinophils in the OVA-induced asthmatic rats were significantly increased compared with those in the normal control group. Mean ± SD, *n* = 9. ^**^ *P* < 0.01 compared to normal control group; ^##^ *P* < 0.01 compared to asthma model group; ^▲▲^ *P* < 0.01 compared to budesonide group

To investigate the function of the co-administration of budesonide and the herbal extracts in OVA-induced inflammatory infiltration in the airways, we examined the cell count in BALF cytospin. As shown in Fig. 3d and e, the number of lymphocytes and eosinophils increased in the BALF of the OVA-induced asthmatic rats compared with those in the normal control group. Only the treatment with the co-adminstration of budesonide and the herbal extracts significantly inhibited the increase in the number of lymphocytes, and each group of drug administration significantly decreased the number of eosinophils, compared with the asthma model group.

IL-4, IL-5 and IgE were closely related to airway variability inflammation. As shown in Fig. 3a, b and c, the content of serum IL-4, IL-5 and IgE were significantly increased after modeling (*P* < 0.01). Compared with the asthma model group, the serum levels of IL-4, IL-5 and IgE in each group of drug administration was remarkably decreased (*P* < 0.01). In addition, there was a significant difference in the level of IL-5 between the groups of budesonide and co-administration.

### Effects of budesonide and the herbal extracts on collagen I and collagen III

**Fig. 4:**
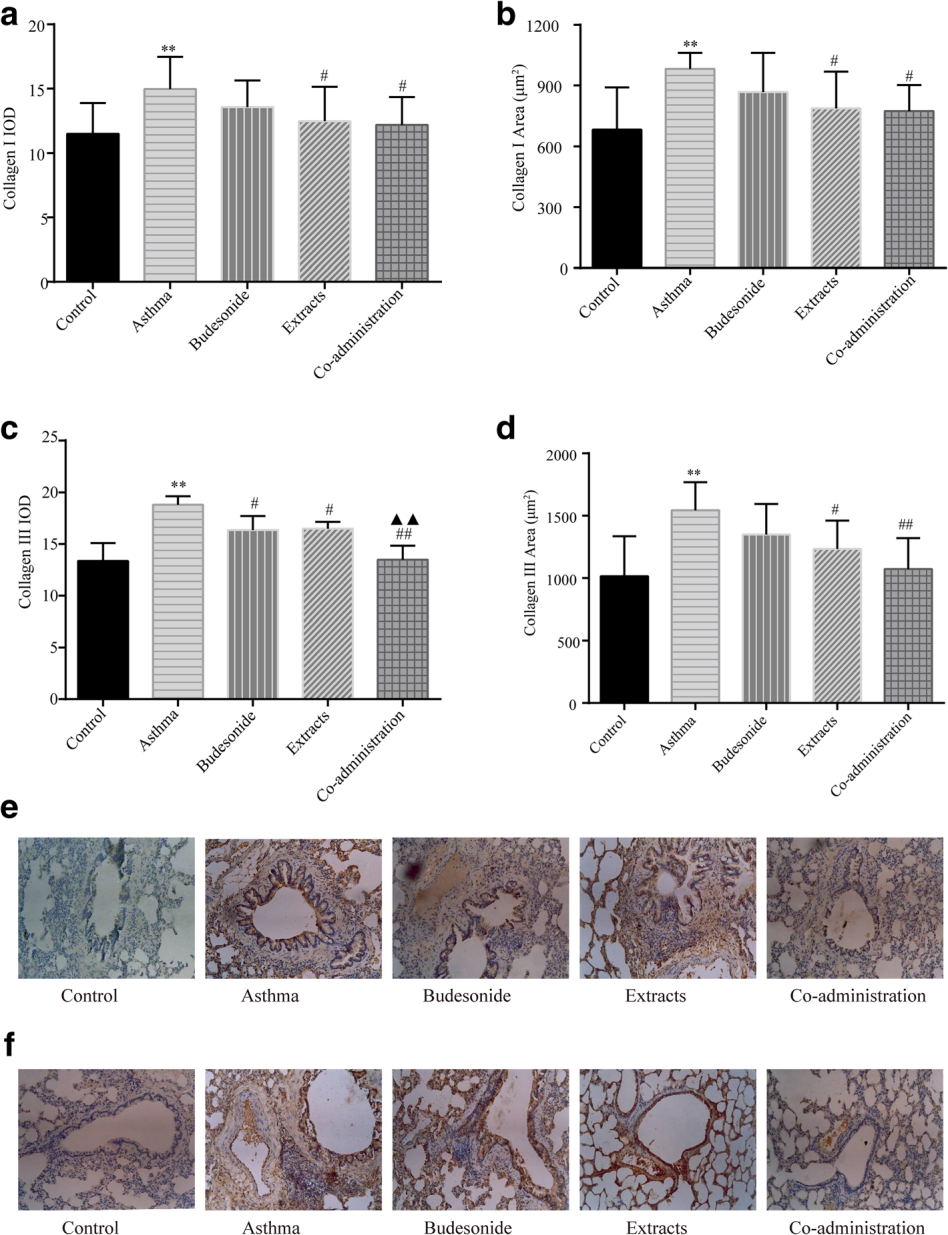
Effects of budesonide and the herbal extracts on expression of collagen I and collagen I. Expression of the two types of collagen in lung tissue was determined by immunohistochemical staining. (**a**) IOD of collagen I. (**b**) Positive area of collagen I. (**c**) IOD of collagen III. (**d**) Positive area of collagen III. (**e**) Representative photomicrographs of collagen I from immunohistochemical-stained lung sections (×400). (**f**) Representative photomicrographs of collagen III from immunohistochemical-stained lung sections (×400). Mean ± SD, *n* = 8. ^**^ *P* < 0.01 compared to normal control group; ^#^ *P* < 0.05 and ^##^ *P* < 0.01 compared to asthma model group; ^▲▲^ *P* < 0.01 compared to budesonide group

Collagen I and collagen III are the most important components of extracellular matrix during collagen deposition in airway remodeling. Immunohistochemical staining results showed that the immunostaining area and IOD of collagen I and collagen III in the asthma group were significantly greater than those in the control group (*P* < 0.01; Fig. 4). Both the extracts group and co-administration group significantly reduced the area and IOD of collagen I and collagen III compared with those in the asthma model group (*P* < 0.05 or *P* < 0.01). There was no significant difference of collagen I expression between the asthma group and the budesonide group. The budesonide group only decreased collagen III IOD (*P* < 0.05). There was a significant difference in collagen III IOD between the budesonide group and the co-administration group (*P* < 0.01), indicating that the combination of budesonide and the herbal extracts had a better effect in reducing collagen III hyperplasia.

### Effects of budesonide and the herbal extracts on TGF-β/Smads

**Fig. 5:**
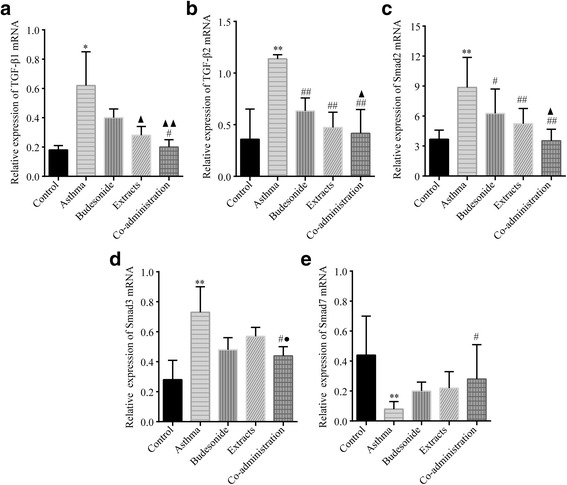
Effects of budesonide and the herbal extracts on mRNA expression of TGF-β/Smads. The mRNA expressions of TGF-β1 (**a**), TGF-β2 (**b**), Smad2 (**c**), Smad3 (**d**) and Smad7 (**e**) in lung tissue were determined by qPCR with β-actin as an internal control. In the asthmatic rats, the mRNA expressions of TGF-β1, TGF-β2, Smad2 and Smad3 were significantly higher, while that of Smad7 was significantly lower than those in the normal group. Mean ± SD, *n* = 7. ^*^ *P* < 0.05 and ^**^ *P* < 0.01 compared to normal control group; ^#^ *P* < 0.05 and ^##^ *P* < 0.01 compared to asthma model group; ^▲^ *P* < 0.05 and ^▲▲^ *P* < 0.01 compared to budesonide group; ^●^ *P* < 0.05 compared to extracts group

It is essential to understand the mechanism of co-administration of budesonide with the herbal extracts for using in clinical development, effectively. In view of recent studies show that TGF-β1 and TGF-β2 play important roles in promoting the structural changes of airway remodeling in asthma [37–39]. The mRNA levels of TGF-β1and TGF-β2 in the five groups were determined by qPCR. Our data showed that the mRNA expressions of TGF-β1 and TGF-β2 in the asthma group were significantly increased compared with the normal control group (*P* < 0.05 and *P* < 0.01). TGF-β1 mRNA expression only in the Co-administration group and TGF-β2 mRNA expression in all the three treatment groups were decreased compared with that in the asthma group (*P* < 0.05 or *P* < 0.01). In addition, there was a significant difference in TGF-β1 or TGF-β2 mRNA expression between the budesonide group and the co-administration group (*P* < 0.01) (Fig. 5a and b).

In order to investigate the expression of active TGF-β signaling in situ, we examined the expression of the intracellular effectors, Smads. An increase in the mRNA expression of Smad2/3 and a decrease in that of Smad7 were observed during prolonged allergen challenge (all *P* < 0.01). All of the treatment groups could considerably decreased Smad2 mRNA expression (*P* < 0.05 or *P* < 0.01). Only co-administration group could considerably altered Smad3 and Smad7 mRNA expression (both *P* < 0.05). There was a significant difference in Smad2 mRNA expression between the budesonide group and the co-administration group (*P* < 0.05). And there was a significant difference in Smad3 mRNA expression among rats treated with the herbal extracts and the co-administration of budesonide and the herbal extracts (*P* < 0.05) (Fig. 5c ~ e).

## Discussion

Asthma is a chronic inflammatory airway disease that is associated with airway remodeling and airway hyperresponsiveness (AHR). Airway inflammation is considered to be the basic pathological change and one of the key pathological mechanisms of repeated attacks of asthma [40, 41], characterized by the infiltration of eosinophils and dominant in Th2 cytokines [4, 5]. IgE is a critical factor for the development of AHR and airway inflammation in asthmatics [42]. And the total IgE level is related to new-onset asthma and the severity of asthma attacks [43, 44]. Allergen-specific Th2 cells are key players in induction and maintenance of allergic asthma. IL-4 is commonly accepted to play a crucial role in driving the differentiation of CD4^+^ Th precursors into Th2-like cells [45]. Apart from determining IgE synthesis by B lymphocytes, IL-4 increases the expression of an inducible form of the low-affinity receptor for IgE (FcεRII/CD23) on B cells [46]. IL-5, another Th2-like cytokines, has profound effects on eosinophil differentiation, activation and survival [47]. The results of the present study show that the number of lymphocytes and eosinophils in BALF and the levels of IL-4, IL-5 and IgE in serum significantly increased in the OVA-induced asthma model rat, while the extracts of HE and FLL, budesonide and the combination of the extracts of HE and FLL with budesonide significantly decreased the levels of IL-4, IL-5 and IgE in serum and the number of eosinophils in BALF. The combined administrations of budesonide with the herbal extracts had better effects on serum IL-5 level than budesonide administration. These results suggest that the combined administrations of budesonide with the herbal extracts might have synergistic effects on airway inflammation.

Airway remodeling is an irreversible airway hyperplasia process that contributes to AHR and irreversible airflow limitation. Airway remodeling is characterized by the increased deposition of collagen in the subepithelial basement membrane region and submucosal layers, smooth muscle hypertrophy and hyperplasia, fibroblast hyperplasia, epithelial metaplasia and goblet cell proliferation [48, 49]. The poor response to treatment observed in patients with refractory asthma may be a consequence of ongoing airway remodeling that result in fixed airway obstruction [50]. We observed that all of the three treatment groups could reduce goblet cell hyperplasia according to the morphometry of PAS-stained lung sections. Only the combination of the extracts of HE and FLL with budesonide significantly decreased collagen deposition according to the morphometry of Masson’s-stained lung sections; meantime inhibited the thickening of airway wall and smooth muscle layer and epithelial hyperplasia according to the morphometry of H&E-stained lung sections. These results suggest that the co-administrations of budesonide with the herbal extracts might have a better synergistic effect on the pathological changes of airway remodeling, as compared to the single use of budesonide or the herbal extracts.

To further confirm that the sub-epithelial area of blue staining measured from Masson’s-stained sections truly reflected an increase in collagen deposition, we also immunostained sections for collagen I and collagen III. Types I and III collagens represent greater than 95% of total lung collagen [51, 52]. Compared with the total blue Masson’s-stained area, the proportional area of immunostained collagen I and collagen III is consistent with the distribution of collagen types in the lung. Quantified analysis of immunohistochemical sections revealed that all of the three treatment groups reduced IOD of collagen III, but the decrease in the co-administration group is significant, as compared to the budesonide group. The extracts group and the co-administration group could depress collagen I IOD and positive area of collagen I and Collagen III. Our study indicates that the co-administration of budesonide and the herbal extracts could prevent and inhibit collagen aggregation of airway remodeling process in allergic airway diseases.

Recently, the TGF-β/Smads signaling pathway was found to be one of the important mechanisms involved in the development of airway remodeling in asthma [53, 54]. Three isoforms of TGF-β are found in mammals, two of which (TGF-β1 and TGF-β2) appear to have critical roles in bronchial asthma. TGF-β1 (most abundant isoform, characteristically associated with endothelial, hematopoietic and connective tissue cells) promotes the expression of profibrotic mediators in various cell types. Furthermore, it is a key component in inducing the epithelial-mesenchymal transition (EMT). In vitro studies have shown that TGF-β1 stimulates the proliferation of mesenchymal cells; moreover, it induces the differentiation of fibroblasts into myofibroblasts to synthesize extracellular matrix (ECM) proteins. Thus, TGF-β1 has the ability to elicit many of the structural alterations of airway remodeling [55, 56]. TGF-β2 (primarily synthesized by airway epithelial cells and neuronal cells) enhances fibroblast activity in the context of inflammatory remodeling in respiratory epithelium. TGF-β2 is secreted in response to epithelial injury [57]. Furthermore, TGF-β2 stimulates collagen synthesis in fibroblasts and is thought to be an important mediator of subepithelial fibrosis. Epithelial-derived TGF-β2 can drive collagen synthesis in subepithelial fibroblasts [58, 59]. As important members of the TGF-β signal transduction system, Smads are the group of intracellular proteins that are critical for transmitting the TGF-β signals from the cell surface to the nucleus to promote transcription of target genes [60]. Among Smads, Smad2 and Smad3 form a heterocomplex with Smad4, and transfer into the cellular nucleus activating DNA transcription to regulate the target gene expression. On the other hand, Smad7 can block the transcription induced by TGF-β through inhibiting its signaling pathway [61, 62]. In our study, we found that treatment with the combinations of the herbal extracts with budesonide reduced the mRNA expression of TGF-β1, TGF-β2, Smad2 and Smad3, while increased Smad7 mRNA expressions. It was reported that there was a link to the expression of TGF-β and the deposition of both collagen I and III in bronchial biopsies, and the expression of both collagen I and III were significantly greater in the subjects with more severe asthma. This effect was not modified following treatment with corticosteroids suggesting that remodeling that had already occurred was not reversible by corticosteroids [63, 64]. So we deduced that the decrease of TGF-β1 and TGF-β2 levels and modulation of the activity of the TGF-β signaling pathway might be a possible mechanism by which the co-administration of budesonide with the herbal extracts inhibits airway remodeling in asthma.

The detailed compositions of both HE and FLL have been previously reported, and mainly include flavonoids, iridoid glycosides and alkaloids, all of which may contribute to the anti-inflammation and anti-remodeling effects of the two herbs. Icariin, identified as the major active ingredient of HE, could exert the anti-inflammatory and anti-tumor functions [65, 66]. Oleanolic acids are the major pharmacologically active components in FLL with anticancer activity, antioxidant activity and immunomodulating effect [67–69]. These results suggest that the anti-inflammatory and anti-remodeling effects of the extracts of HE and FLL may be the result of cooperative actions among multiple compounds with their multiple components working together to common affects. The exact roles of the different active components when used for the treatment of asthma remain to be further investigated.

## Conclusions

In summary, our study demonstrated that inhaled GC (budesonide) combined with the extracts of HE and FLL has a better synergistic effect on the airway remodeling in asthma rats, compared with the single use of budesonide or the herbal extracts. It was deduced that the mechanisms of anti-remodeling in asthma airway involve in inhibiting the mRNA expression of TGF-β1 and TGF-β2 as well as modulation of TGF-β signaling in the lung tissue. Additional studies exploring the mechanism of the systemic therapy are needed.

## Abbreviations

AHR: Airway hyperresponsiveness
ANOVA: One-way analysis of variance
BALF: Bronchoalveolar lavage fluid
cDNA: Complementary DNA
ECM: Extracellular matrix
EMT: Epithelial-mesenchymal transition
FLL: Fructus Ligustri Lucidi
GC: Glucocorticoid
GR: Glucocorticoids receptor
H&E: Hematoxylin and eosin
HE: Herba Epimedii
HPA: Hypothalamic-pituitary-adrenal
IL: Interleukin
IOD: Integral optical density
*ip*: Intraperitoneal
OVA: Ovalbumin
PAS: Periodic-acid schiff
Pbm: Perimeter of basement membrane
qPCR: Quantitative Real-Time PCR
RQ: Relative quantification
SD: Standard deviation
TCM: Traditional Chinese Medicine
TGF: Transforming growth factor
Th: Helper T cells
Wam: Area of smooth muscle
Wat: Total area of airway wall
